# A 3-month-old child with COVID-19: A case report

**DOI:** 10.1097/MD.0000000000020661

**Published:** 2020-06-05

**Authors:** Chenxi Li, Fan Luo, Bing Wu

**Affiliations:** a Department of Radiology, Public Health Clinical Center of Chengdu; b Department of Radiology, West China Hospital, Sichuan University, Chengdu, China.

**Keywords:** chest computed tomography, children, COVID-19, SARS-CoV-2

## Abstract

**Introduction:**

Coronavirus disease 2019 (COVID-19) is pandemic and is a medical issue. However, children account for a small portion of those with the disease, and there are few published reports of COVID-19 in children. The patient reported in this case report is the youngest case reported in Chengdu, China to date.

**Patient concerns:**

A 3-month-old male infant presented with cough and rhinorrhea.

**Diagnosis:**

Family members from Wuhan, the epicenter of the epidemic came to stay in the patient's home 16 days before the onset of his disease, and his mother had been diagnosed with COVID-19. He was diagnosed with COVID-19 based on a history of exposure and severe acute respiratory syndrome coronavirus 2 (SARS-CoV-2), detected using reverse transcription polymerase chain reaction (RT-PCR).

**Interventions:**

The patient was admitted to hospital and treated symptomatically with oral medication.

**Outcomes:**

The patient recovered completely and was discharged after one month of hospitalization. He tested negative for SARS-CoV-2 using RT-PCR and a chest CT performed 4 weeks after admission showed marked improvement prior to discharge.

**Conclusion:**

Clinicians must be aware of the presentation of COVID-19 in children because it differs from that in adults.

## 1 Introduction

The population is generally susceptible to severe acute respiratory syndrome coronavirus 2 (SARS-CoV-2), but according to reports, children under 10 years of age only account for 0.35% of cases in China.^[1]^ The lack of pediatric cases results in difficulty in making a clinical diagnosis in children. This report describes the case of a 3-month-old child, who is the youngest patient treated for SARS-CoV-2 infection in Chengdu so far. We describe the clinical features, laboratory results, computed tomography (CT) images, and treatment in order to provide information for clinicians who manage children with SARS-CoV-2 infection.

## 2 Case presentation

**Figure 1:**
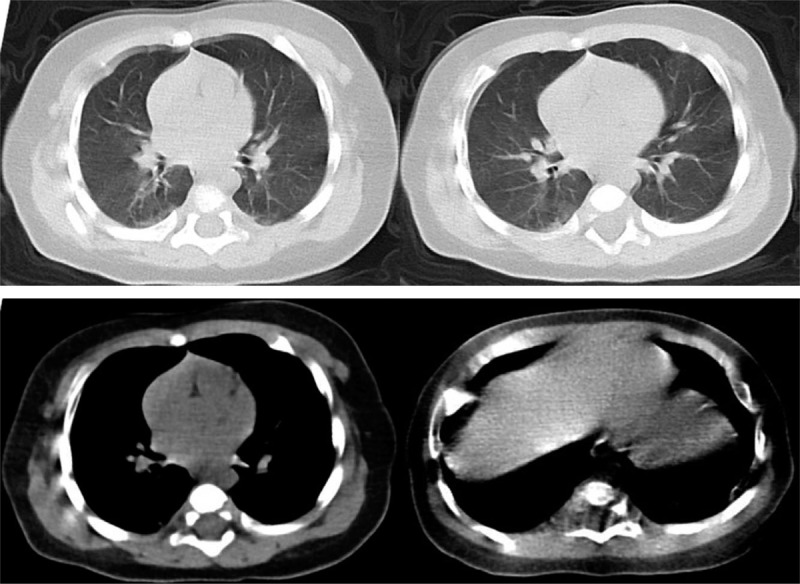
Unenhanced chest CT taken on admission to hospital showing small nodules in the bilateral upper lobe and the dorsal segment of the lower lobe; mainly distributed along the subpleural area; bronchovascular bundles thickening. There is no pleural effusion or lymphadenopathy.

A 3-month-old male infant presented with a non-productive cough and rhinorrhea with no apparent cause on February 2, 2020. He had no fever, vomiting, or diarrhea. His condition had not improved after he was given a “common cold medication” by family members. A CT image taken in a local hospital showed “nodules and patchy opacification in the middle lobe of the right lung, the lingual segment of the upper lobe of the left lung, and the lower lobes of both lungs, predominantly in subpleural area, possibly viral pneumonia.” Because the patient's mother had confirmed COVID-19, the center for disease control (CDC) arranged SARS-CoV-2 testing using reverse transcription polymerase chain reaction (RT-PCR) assays immediately. The result was positive, so the patient was referred to our hospital for isolation and treatment. He had shown no signs for 16 days after close contact with his relatives from Wuhan, so we speculated that the incubation period was 16 days. The main clinical features were cough and rhinorrhea, but the patient did not have a fever or digestive problems. Laboratory tests showed an elevated white blood cell (WBC) count and lymphocyte count, decreased neutrophil count, low C-reactive protein (CRP), and elevated lactate dehydrogenase (LDH), alanine aminotransferase (ALT), and aspartate transaminase (AST). In addition, the creatine kinase myocardial band (CK-MB), myoglobin, and troponin T-hypersensitivity were abnormal, and the test for *Mycoplasma pneumoniae* was negative (Table 1). An unenhanced chest CT showed “small nodules in the bilateral upper lobe and the dorsal segment of the lower lobe; mainly distributed along the subpleural area; bronchovascular bundles thickening; no pleural effusion or lymphadenopathy” (Fig. 1). After the symptomatic treatment for 3 weeks, including the use of ambroxol hydrochloride oral solution (2 ml, 2 times a day), fructose sodium diphosphate oral solution (3 ml, 3 times a day) and Ganxile granules (a traditional Chinese liver tonic, 1.33 g, 3 times a day), the patient showed great improvement on his second CT images 4 weeks later. His RT-PCR test turned to negative 1 month after admission and he was discharged after a complete recovery.

**Table 1:**
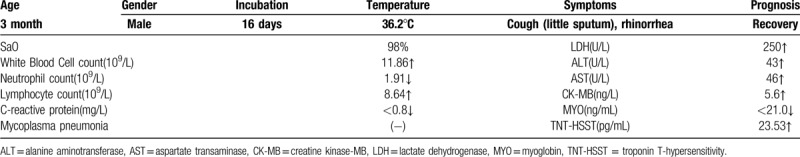
Patient's clinical manifestation and laboratory results.

## 3 Discussion

The infant lived in Chengdu in China with relatives who had returned from Wuhan, in Hunan Province, which was the epicenter of the epidemic at the time, and had close contact with them for several days. His mother had been confirmed with COVID-19, so the epidemiological link was evident. The incubation period of COVID-19 is usually 1 to 14 days, with an average of 5.2 days,^[2]^ but the patient only became ill 16 days after his relatives had returned from Wuhan, which is longer than the upper limit, but the patient's family members’ may not have noticed his respiratory condition immediately the patient was an infant. Wang et al^[3]^ studied 34 pediatric cases of COVID-19. They reported that children often have mild, atypical disease, with fever (50%) and cough (38%) being the main presenting complaints. This patient presented with cough and rhinorrhea, but did not have a fever, so the clinical features differed from previous reports. The Chinese national guidelines for the diagnosis and treatment of COVID-19^[4]^ state that it is common for patients to have a normal or decreased WBC count, a and decreased neutrophil count; that some patients have increased LDH, and myoglobin; and that most patients have an elevated CRP. However, this patient had an increased WBC count and lymphocyte count, combined with a decreased CRP, so his laboratory findings were also atypical. Moreover, the increased LDH, ALT and AST indicate liver damage, while abnormal cardiac enzymes indicate myocardial damage. The patient's respiratory problems resolved and his liver and myocardial function markers returned to normal after treatment.

A previous study^[5]^ reviewed the CT images of the lungs of 22 children with COVID-19, and found that lesions could involve all lobes; lesions in the upper lobes are more severe; and there are 4 categories of CT findings:

(1) pure ground-glass opacities (GGOs);
(2) pure consolidation;
(3) both GGOs and consolidation; and
(4) small nodules and patches along with bronchovascular bundles, similar to bronchopneumonia.

The study also found that pleural effusion and lymphadenopathy were uncommon in CT images. The chest CT of this patient showed bronchovascular bundle thickening, small nodules and patchy opacification, distributed along with the bronchovascular bundles or subpleural area, with no sign of pleural effusion or lymphadenopathy, which corresponds to the fourth category mentioned above. The CT features in children differ somewhat from those of adults. In adults, GGOs are typically present, and lesions are distributed predominantly in the peripheral and posterior areas of both lungs with a more extensive range, creating the common “reverse butterfly sign.”^[6]^ Furthermore, children tend to have milder disease than adults. COVID-19 case severity in children is classified as mild (asymptomatic or subclinical), normal, severe, and critical.^[7]^ Most cases in children are of mild or normal severity. The case fatality rate in children is very low. The milder course of the disease in children may relate to their immature immune system, which does not overreact to the virus, but the reasons why disease tends to be less severe in children requires further research. A recent study^[8]^ found that some children may develop to severe disease and experience an adverse outcome, especially when atypical and mild presentations delay the diagnosis. The first severe pediatric case reported was that of a 13-month-old boy in Wuhan with no previous comorbidity who developed shock, acute respiratory distress, and renal failure.^[9]^ The risk factors for severe disease in children is still uncertain due to the small number of severe cases reported in children. Additional pediatric cases need to be described, and case series need to be subjected to detailed statistical analysis. Our patient fell within the normal severity category; his respiratory abnormalities were mild, but his blood tests showed evidence of liver and myocardial injury, which improved after treatment. The clinician must be mindful of the changes in other organ systems beside the respiratory system in the management of children with COVID-19. Several laboratory figures disagree with the recommendation.^[4]^ It may be because most patients in previous studies have been adults, and there are differences between children and adults in how they respond to infection. In patients with atypical CT features, it is necessary to consider other infectious diseases of the lungs in the differential diagnosis, including bronchopneumonia, mycoplasma pneumonia, and seasonal influenza, especially when there is no history of exposure to SARS-CoV-2. Lastly, it is necessary to review more pediatric cases and to do further research on the CT manifestations in children in order to improve the radiological diagnosis, thereby enabling early and targeted treatment.

## 4 Disclosure

This study was approved by the institutional review board of the Public Health and Clinical Center of Chengdu. The requirement for informed consent was obtained from the patient's parents. There is no conflict of interest exists in the submission of this manuscript, and manuscript is approved by all authors for publication.

## Acknowledgments

We wish to express our sincere thanks to Yujie Yuan for his help for language and constructive suggestions for this article.

## Author contributions

FL collected and recorded the original data, CXL analyzed the case and was a major contributor in writing the manuscript, BW guided all the work.
